# Constructing rapid water vapor transport channels within mixed matrix membranes based on two-dimensional mesoporous nanosheets

**DOI:** 10.1038/s42004-022-00681-9

**Published:** 2022-05-24

**Authors:** Fengkai Wang, Shurui Han, Yanli Zhang, Lei Gao, Xu Li, Lizhi Zhao, Hui Ye, Hong Li, Qingping Xin, Yuzhong Zhang

**Affiliations:** State Key Laboratory of Separation Membranes and Membrane Processes, School of Materials Science and Engineering, Tiangong University, Tianjin, 300387 China

**Keywords:** Two-dimensional materials, Two-dimensional materials, Liquid crystals, Organic-inorganic nanostructures

## Abstract

Membrane technology is an effective strategy for gas dehumidification and fuel cell humidification. In this study, cerium fluoride oxide (F-Ce) two-dimensional (2D) mesoporous nanosheets and their composite with 1-ethyl-3-methylimidazolium dicyanamide ([Emim][DCA]) ionic liquids (ILs) (IL@F-Ce) are introduced as fillers into polyether block amide (PEBAX® 1074) to fabricate mixed matrix membranes (MMMs). The slit-shaped mesoporous structure of the nanosheets facilitates the construction of water vapor rapid transport channels in MMMs. The permeability and selectivity of water vapor for MMMs loaded with F-Ce nanosheets are greatly improved, and the performance of MMMs loaded with IL@F-Ce nanosheets are much better than the former. Particularly, the MMM with IL@F-Ce content of 4 wt.% achieves the highest H_2_O permeability of 4.53 × 10^5^ Barrer, which is more than twice that of the pure PEBAX membrane, and the selectivity is increased by 83%. Thus, the MMMs based on 2D mesoporous nanosheets have considerable potential application in industrial-scale dehydration and humidification processes.

## Abstract (web summary)

Gas dehumidification with permeable membranes is typically a technique low in operating costs and environmental impact, making it preferable over condensation or adsorption methods, but membrane permeability and selectivity remain a trade-off. Here, the authors use cerium fluoride oxide mesoporous nanosheets in the ionic liquid [Emim][DCA] as a filler in the polyether block amide PEBAX to achieve a mixed matrix membrane with a water vapour permeability of 4.53 × 10^5^ Barrer and a H_2_O/N_2_ selectivity of 1.69 × 10^5^.

## Introduction

With the development of economics and technology, gas dehumidification and humidification has become an important component in daily life and production^1^. Moisture is ubiquitous in production and life, and direct emission of moisture will result in a great waste of water resources. If the dehumidification efficiency of these part of the humid gas can be improved and the condensed water can be efficiently recovered, it will be a significant help to water recycling in water-deficient areas. The traditional dehumidification methods include low-temperature condensation^2^, liquid/solid adsorption^3–5^, and electrostatic dehydration^6,7^. However, there are non-negligible disadvantages such as high energy consumption, serious pollution, large occupied area and maintenance difficulties in the above technologies. Compared with traditional gas dehumidification methods, the membrane gas dehumidification process can be carried out continuously, and therefore has the advantages of simple operation, low operating costs and easy maintenance^8^. Water vapor removal is mainly used in flue gas dehumidification^9^, natural gas dehydration^10,11^, and compressed air drying^12^. Furthermore, In a fuel cell system, the proton membrane can only conduct hydrogen ions under a certain humidity. Low humidity in the proton membrane will cause the internal resistance of the battery to increase, thereby greatly reducing the operating voltage of the cell. On the other hand, excessive water content can block or even damage the stack. At present, membrane humidifiers have been implemented in proton exchange membrane fuel cell systems to humidify the cathode air before it enters the stack, and the gas-to-gas humidification method can make full use of the water and heat in the exhaust gas without causing water blockage.

The membrane material is the core of gas humidification and dehumidification technology. In addition to having both high permeability and selectivity, the ideal membrane material should also have the advantages of high mechanical strength, high corrosion resistance, good stability and low cost. The hydrophilic membrane is widely chosen for gas humidification and dehumidification technology. The strong interaction between water molecules and hydrophilic membrane makes the membrane have high water vapor solubility and diffusivity^13,14^, the most widely used humidification and dehumidification membrane in the market is perfluorosulfonic acid resin (PFSA), which has high chemical stability and mechanical strength at high humidity, and the sulfonic acid group contained in its the molecular structure provide good hydrophilicity^15,16^. According to reports^17,18^, the adsorption of water can swell the hydrophilic region in the PFSA membrane, and thus a diffusion pathway for water molecules produces. Afterwards, the water phases are taken away by dry purge gas. However, its further commercial application is restricted due to its high cost. In addition, other hydrophilic membranes, including sulfonated polyether ether ketone (SPEEK)^19,20^, sulfonated polyethersulfone (SPES)^21^, and PEBAX^®^ 1074^9,22^ have also been widely studied. SPEEK and SPES can be obtained by the sulfonation of polyether ether ketone and polyether sulfone, respectively, and they exhibit excellent performance in the fields of humidification and dehumidification. The performance of SPEEK and SPES-based membranes is related to the degree of sulfonation. The introduction of sulfonic acid groups in the polymer increases steric hindrance, resulting in a decrease in the flexibility of the molecular chain. At the same time, when the degree of sulfonation increases, the water absorption of the membrane increases, but the swelling resistance decreases^23^. Therefore, the degree of sulfonation is an important indicator for selecting SPEEK and SPES. PEBAX^®^ 1074 is a commercial hydrophilic block copolymer, which is composed of 45 wt.% hard polyamide block (PA12) and 55 wt.% amorphous PEO block. The solubility and diffusion of vapor and gas mainly occur in the PEO phase in the polymer, while the PA12 segments deliver excellent mechanical stability to the membrane. Potreck et al.^22^ used PEBAX^®^1074 as a matrix to prepare a homogeneous membrane and perform water vapor adsorption and H_2_O/N_2_ separation measurements on it, they found that the PEBAX^®^ 1074 polymer material can efficiently adsorb water vapor and display extremely high selectivity for N_2_.

Searching for low-cost alternative membranes is a major challenge in the membrane dehumidification and humidification field. Mixed matrix membranes (MMMs) combine the advantages of fillers and polymer matrix with good permeability, mechanical strength, and processing properties, and have been applied to the study of gas humidification and dehumidification in recent years. Ingole et al.^24^ introduced carboxylated titanium dioxide (C-TiO_2_) and hydroxylated titanium dioxide (H-TiO_2_) nanoparticles into a polyamide/polyester layer, which could considerably improve the water vapor permeability and selectivity of the membrane. The maximum water vapor permeability of C-TiO_2_ nanoparticles loaded MMMs reached 1131 GPU, and the H_2_O/N_2_ selectivity reached 548. Akhtar et al.^25^ prepared MMMs by embedding graphene oxide nanosheets into a hydrophilic microphase-separated block copolymer and found that the water vapor permeability decreased by 12% and the selectivity increased by 8-fold. MMMs containing 13X zeolite particles were prepared by Wolinska-Grabczyk et al.^26^, and the water vapor permeability of the membrane was significantly improved, which was ascribed to the introduction of zeolite enhancing the water solubility of the membrane. Bounos et al.^27^ fabricated MMMs by mixing isotactic polypropylene (i-pp) with multiwalled carbon nanotubes (MWCNTs), resulting in increased water permeability. Baig et al.^28^ used graphene oxide (GO) and GO-TiO_2_ as fillers to prepare nanocomposite membranes, and the introduction of fillers provides active sites for water molecules, thereby significantly improving the permeability of water vapor. Akhtar et al.^29^ prepared PBI/ TiO_2_ MMMs, and the results showed the water vapor permeability and H_2_O/N_2_ selectivity have almost doubled compared with the pure PBI membrane. In summary, the existence of these fillers mainly improves the water vapor permeability and selectivity by enhancing the hydrophilicity of the membrane.

Recently, ionic liquids have been widely used to capture water vapor due to their extreme hygroscopicity. Kudasheva et al.^30^ prepared three supported liquid membranes composed of ionic liquids., demonstrating that these ionic liquids have a high adsorption effect on water. However, these supported ionic liquid membranes (SILM) have poor stability. Park et al.^31^ used a combination of ILs and metal-organic frameworks (MOFs) to solve this problem, showing improved water vapor permeability and selectivity. In our previous work, the morphology, structure, growth mechanism, and stability of F-Ce nanosheets have been studied. The F-Ce represents a 2D nanosheet composed of F-Ce one-atom-layers and acetate anions. Compared with traditional nanoparticles, F-Ce nanosheets have the advantages of mesoporous structure, hydrophilic surfaces, high surface area, and ultrathin morphology (1–2 nm in thickness). F-Ce nanosheets and their composite with ILs have been introduced into the polymer matrix for effective CO_2_ removal applications^32^, and the results show that MMMs doped with F-Ce nanosheets have good properties for CO_2_/CH_4_ separation, and exceed the Robeson 2008 upper bound line.

Herein, we incorporate F-Ce nanosheets and functionalized F-Ce nanosheets (IL@F-Ce) as a filler in the PEBAX^®^ 1074 polymer matrix. The hydrophilic surface of F-Ce nanosheets facilitates the adsorption of water molecules. Additionally, the slit-shaped mesoporous structure of F-Ce nanosheets provides a rapid transport channel for water molecules. ILs are used to modify the surface and pores of F-Ce nanosheets, providing more adsorption sites for water molecules and constructing well-defined water molecule transport channels. we studied the effects of loadings and pore diameters of the 2D mesoporous nanosheets on the morphology, physical, and water vapor permeability properties of MMMs. For comparison, the effects of nanosheets on SPEEK and Nafion matrix materials are explored. This study may provide important insights for air dehumidification and proton exchange membrane humidification.

## Results

### Structure and morphology of the 2D mesoporous nanosheets

**Fig. 1:**
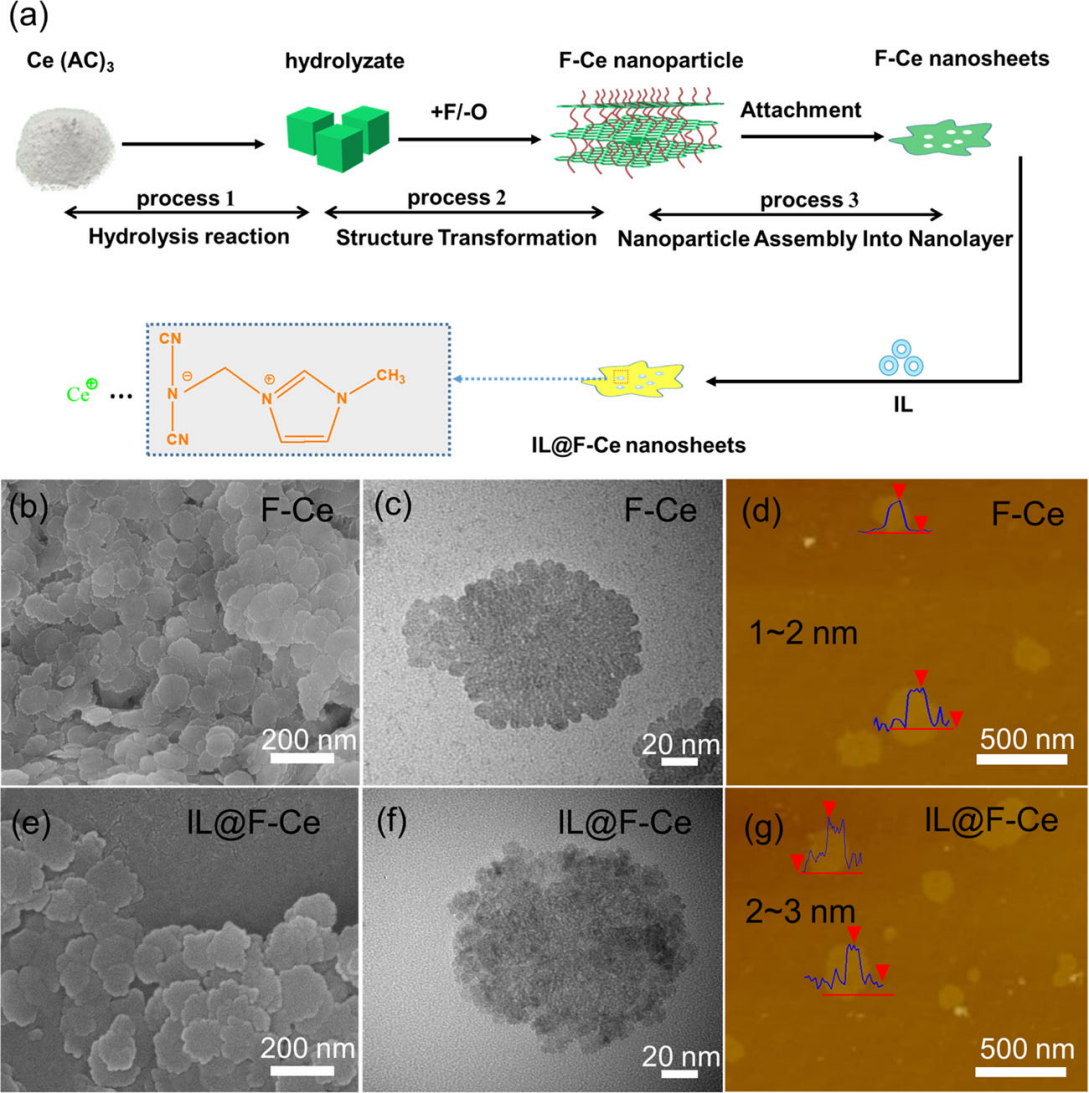
Formation mechanism and morphological characterization of F-Ce and IL@F-Ce nanosheets. **a** The plausible formation mechanism of F-Ce and IL@F-Ce nanosheets. **b, e** SEM images, **c, f** TEM images, **d, g** AFM images and thickness of F-Ce and IL@F-Ce nanosheets.

2D mesoporous nanosheets were synthesised by introducing fluorine ions into the aqueous solution of cerium acetate, and then they are functionalized with ILs obtaining IL@F-Ce nanosheets (Fig. 1a). The IL@F-Ce nanosheets (Fig. 1e) have a regular 2D morphology and show jagged disc features with particle sizes in the range of 160–200 nm. The morphology is similar to those of F-Ce nanosheets (Fig. 1b) with no apparent modification. The TEM images (Fig. 1c, f) can be observed that the surface structure of IL@F-Ce nanosheets is slightly harsher than that of pristine F-Ce nanosheets, which is caused by the introduction of ILs. Some areas on the nanosheets have lower background contrast, indicating that the nanosheets may be mesoporous nanosheets (The specific pore size will be studied later). AFM technology is manipulated to analyze the thickness of 2D mesoporous nanosheets as illustrated in Fig. 1d, g. Results show that the IL@F-Ce nanosheets are layered structures similar to that of F-Ce nanosheets, and the thickness is increased to 2–3 nm.

**Fig. 2:**
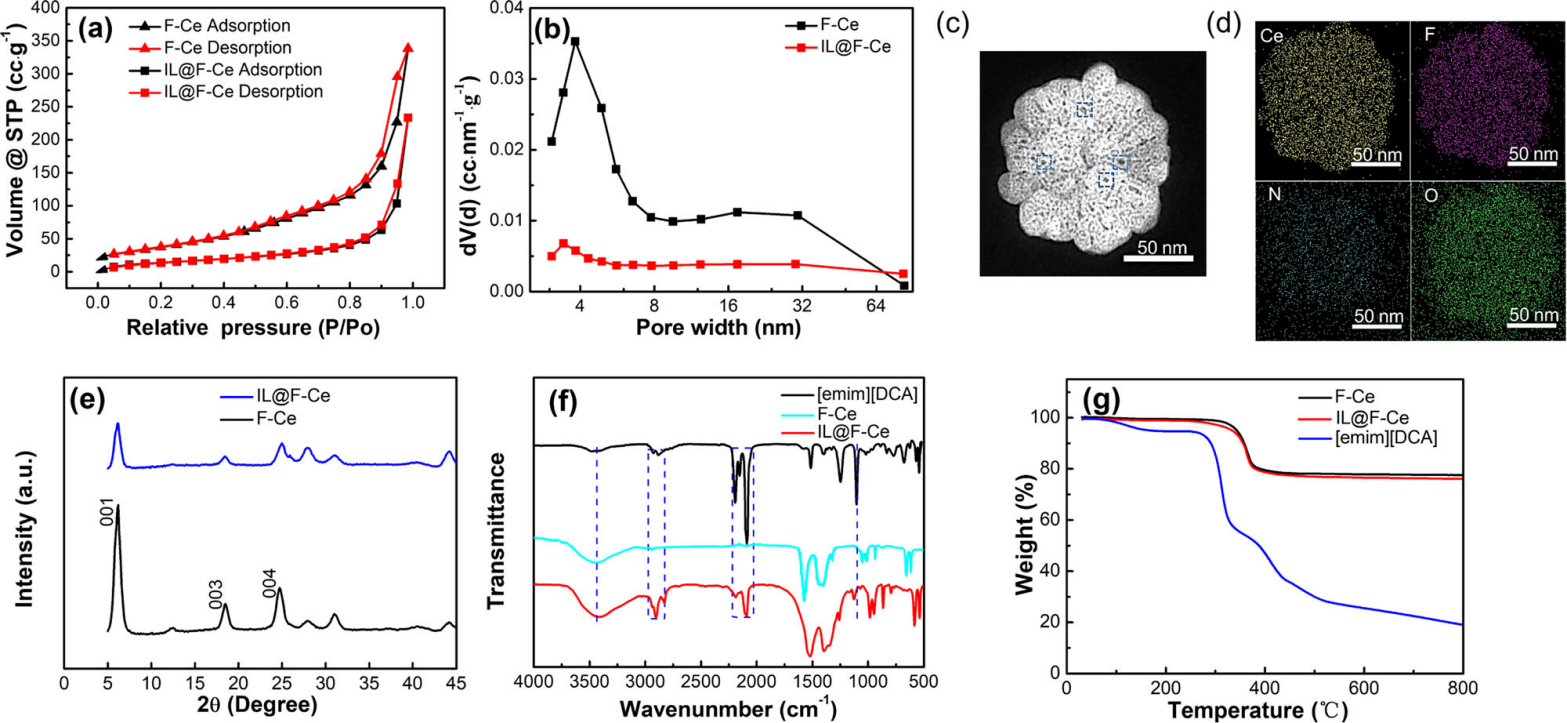
Structural analysis of F-Ce and IL@F-Ce nanosheets. **a** N_2_ adsorption-desorption isotherm, and **b** pore diameters distribution of F-Ce and IL@F-Ce nanosheets. **c** HAADF-STEM image and **d** EDS mappings of IL@F-Ce nanosheets, **e** XRD patterns, **f** FT-IR curves, and **g** TG curves of F-Ce and IL@F-Ce nanosheets.

The N_2_ adsorption measurement shows that both F-Ce and IL@F-Ce nanosheets exhibit type IV adsorption behavior (Fig. 2a), indicating that the insertion of ILs does not significantly impact the mesoporous properties of F-Ce nanosheets. The specific surface area and pore volume of F-Ce nanosheets are 162.75 m^2^·g^−1^ and 0.52 cm^3^·g^−1^, respectively. On the basis of the BJH model (Fig. 2b), F-Ce nanosheets have an average pore diameter of 3.81 nm. The specific surface area (53.17 m^2^·g^−1^), pore volume (0.36 cm^3^·g^−1^), and average pore diameter (3.41 nm) of IL@F-Ce nanosheets are decreased with the loading of ILs. Results show that F-Ce nanosheets have a mesoporous structure, and the ILs occupy part of the pores for the nanosheets. The slit-shaped pore structure of nanosheets can be seen from the HAADF-STEM image in Fig. 2c (noted by the blue dotted circles). The EDS spectrometer is used to characterize the distribution of the F, Ce, N, and O elements on the IL@F-Ce nanosheet (Fig. 2d). The N elements in IL@F-Ce from the ionic liquid are evenly distributed in the nanosheets, indicating that the ionic liquid is loaded on the nanosheets relatively uniformly. XRD and FT-IR are conducted to verify the physical structure changes of the nanosheets. XRD curves are exhibited in Fig. 2e, and the characteristic peak for F-Ce still exists after the [EMIM][DCA] is incorporated. The (001), (003), and (004) peaks of IL@F-Ce become weak because the introduction of ionic liquid reduces the crystallinity of F-Ce nanosheets and the morphology changes, which is consistent with the results of TEM. The FT-IR spectra of [EMIM][DCA], F-Ce, and IL@F-Ce nanosheets as displayed in Fig. 2f. For F-Ce nanosheets, the strong vibration peaks at 1444 and 1576 cm^−1^ are derived from the symmetric and asymmetric stretching vibrations of the C=O double bond, respectively. The strong and narrow frequency bands between 900 and 985 cm^−1^ are correlated with the C-C symmetric stretching vibration of acetate anion, while the vibration region between 500 and 710 cm^−1^ is mainly ascribed to the in-plane and out-of-plane bending vibration of O=C-O. The spectrum of IL@F-Ce nanosheets displays new peaks at 2240, 2140, 1335, and 701 cm^−1^, respectively. These peaks are attributed to the vibration of the [DCA] anion, which represents the stretching and antisymmetric stretching vibrations of the C≡N bond and the in-plane symmetric and asymmetric deformation of the C-N-C bond, respectively^33^. The peak at 2963 cm^−1^ results from the stretching vibration of the C-H bond on the cationic imidazole ring^34^. The appearance of these characteristic peaks indicates that [EMIM][DCA] is successfully introduced onto F-Ce. Particularly, the blue shift of the C-N-C bond (from 1335 to 1345 cm^−1^) in IL@F-Ce indicates a strong interaction between ILs and F-Ce nanosheets. In addition, the characteristic peak of 3400 cm^−1^ is related to the stretching vibration of -OH in a water molecule, indicating that the surface of 2D mesoporous nanosheets has a good adsorption effect on water molecules.

Elemental analysis from XPS of F, Ce, O, and N are used to quantify the ILs loading in the IL@F-Ce composite fillers. As can be seen from Supplementary Table 1, after washing treatment, the ratio of Ce/N elements in IL@F-Ce remains unchanged, indicating that the washing process effectively removes the ILs that do not participate in the reaction. The ILs content of IL@F-Ce composite is calculated to be 4wt% from XPS data. The thermal stability of IL@F-Ce is illustrated in Fig. 2g, and the slightly reduced thermal stability of IL@F-Ce indicates a strong interaction between [EMIM][DCA] and F-Ce 2D nanosheet.

### Structure and morphology of the MMMs

**Fig. 3:**
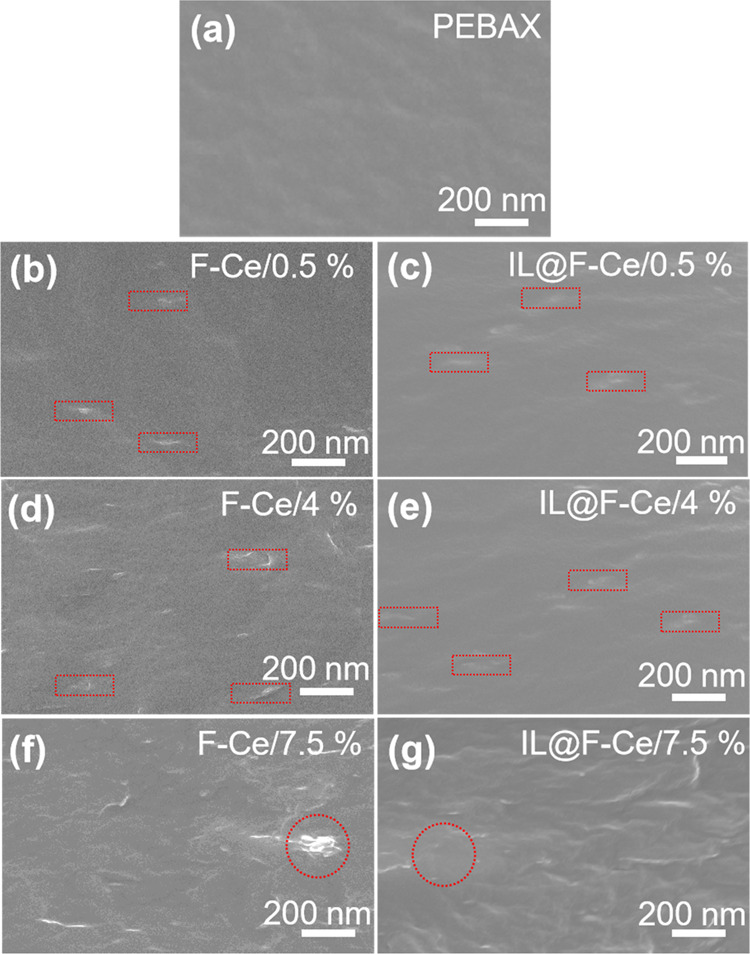
Morphologies of MMMs. Cross-sectional SEM images of the **a** pure PEBAX membrane and MMMs loaded with **b** 0.5 wt.%, **d** 4 wt.%, and **f** 7.5 wt.% F-Ce nanosheets and **c** 0.5 wt.%, **e** 4 wt.%, and **g** 7.5 wt.% IL@F-Ce nanosheets.

The SEM images of MMMs with 2D mesoporous nanosheets contents of 0.5wt%, 4wt%, and 7.5wt%, respectively, are shown in Fig. 3. The cross-sectional image of the pure PEBAX membrane (Fig. 3a) is dense. The 2D mesoporous nanosheets are homogeneously mixed with the PEBAX matrix under a loading amount of 4wt % (Fig. 3d, e). The cross-sectional image of MMMs with apparent filler aggregations with the increase in nanosheets content up to 7.5wt% (Fig. 3f, g). The distribution of Ce, N, and O elements in the MMM at nanosheets content of 4wt% is characterized by an EDS spectrometer. As shown in Supplementary Fig. 1, the Ce, O, and N elements are regularly distributed in the MMM, indicating that the nanosheets at a content of 4wt% have excellent dispersibility in the PEBAX matrix and display good compatibility with the matrix.

**Fig. 4:**
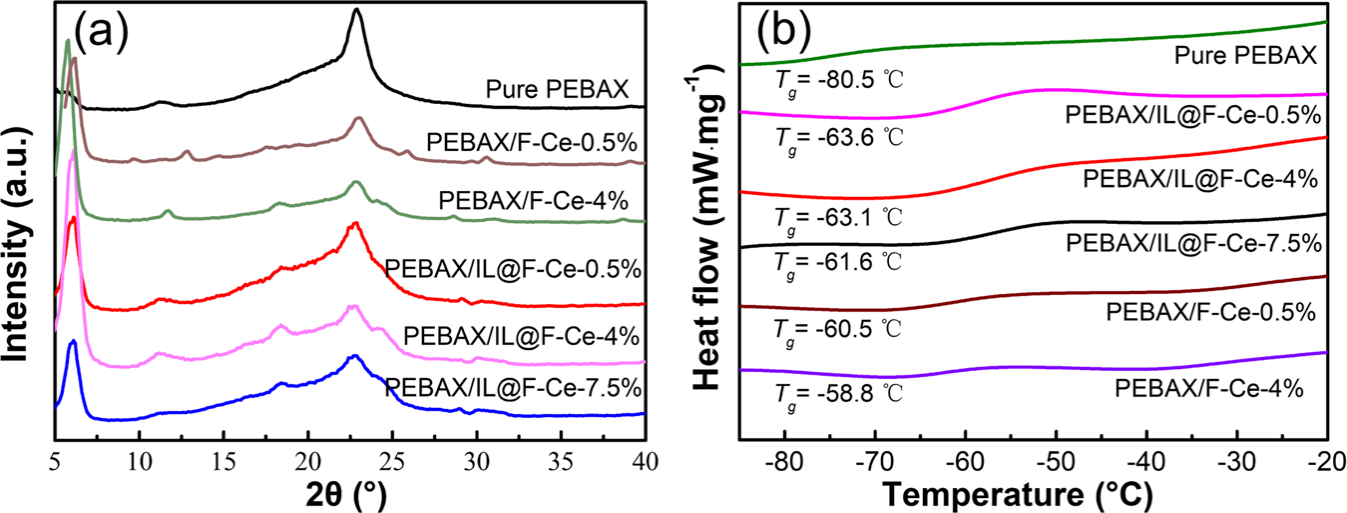
Structural analysis of MMMs. **a** XRD patterns, and **b** DSC thermograms of the pure PEBAX membrane and MMMs.

In the XRD patterns (Fig. 4a), the PEBAX membrane presents a broad characteristic peak at around 2θ = 23°, which is attributed to the soft PEO segment. After loading of 2D mesoporous nanosheets, the characteristic peak of PEBAX at around 23° is weakened, and the crystallinity is slightly decreased. As the content of nanosheets increases, the characteristic diffraction peaks at 2θ = 6.2°, 18°, and 28.8° become more obvious. It shows that the structure of F-Ce nanosheets is not damaged during the preparation of MMMs, and the crystal structure is maintained well. The characteristic peaks of PEBAX/F-Ce-4% and PEBAX/ IL@F-Ce-4% membrane are higher than those of other MMMs, indicating that the interaction between the nanosheets and the polymer is strong and the crystallinity remains well. The DSC results are shown in Fig. 4b. The T_g_ of MMMs is lower than that of the pure PEBAX membrane, signifying that the loading of 2D nanosheets limits polymeric chain packing and increases the flexibility of the PEBAX segment, thereby generating more free volume for gas molecule transfer, which may help to improve gas diffusion.

The water contact angle of MMMs loaded with different 2D mesoporous nanosheets contents is displayed in Supplementary Fig. 2. The contact angle of MMMs first decreases progressively with the increase of F-Ce nanosheets contents and then remains basically unchanged. The contact angle of the pure PEBAX membrane is 67.28°, and the water contact angle is 60.22° as the loading of F-Ce nanosheets is 4%, which is 10% lower than that of the PEBAX membrane. It’s mainly because the surface of F-Ce contains rich hydrophilic functional groups (-COOH), which help to improve the hydrophilic properties of the membrane. Besides, with increasing surface roughness (Supplementary Fig. 3), the contact angle decreases for MMMs. The water contact angle is reduced by 13% compared with the PEBAX membrane as the loading amount of IL@F-Ce nanosheets is 4%. This may be because of the introduction of [DCA] anions (See Supplementary Fig. 11 for structures) in the modified nanosheets, which further improves the hydrophilicity of the membrane. These nanosheets have an important role in improving the hydrophilicity of the membrane, which can affect the water vapor permeability of the membrane.

### Water vapor adsorption and diffusion of MMMs

**Fig. 5:**
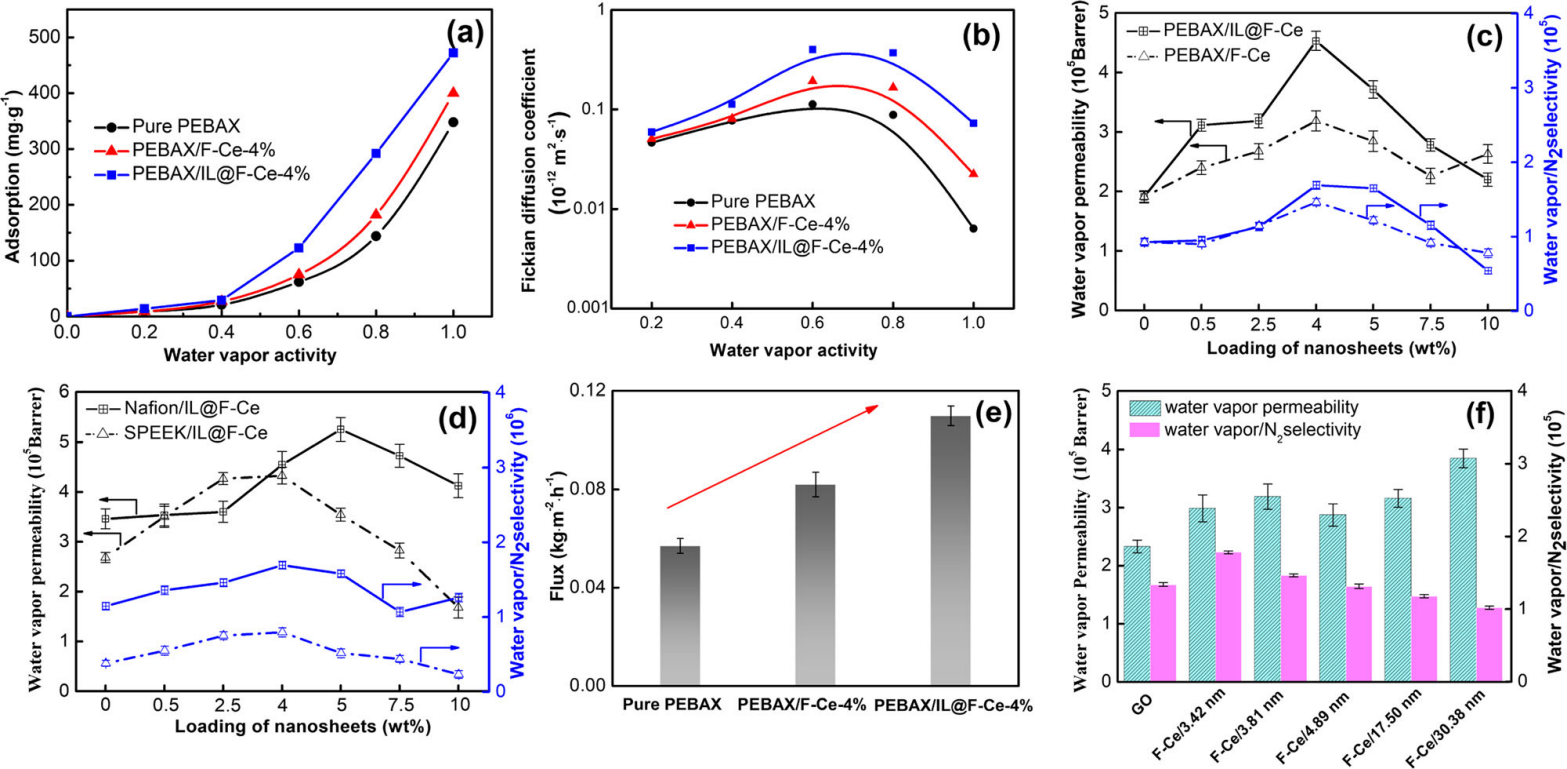
Water vapor permeation performance. **a** Adsorption and **b** diffusion in pure PEBAX, PEBAX/F-Ce-4%, and PEBAX/IL@F-Ce-4% membrane at different water vapor activities. The water vapor permeability and H_2_O/N_2_ selectivity of the (**c**) PEBAX/F-Ce MMMs and PEBAX/IL@F-Ce MMMs, **d** Nafion/IL@F-Ce MMMs and SPEEK/IL@F-Ce MMMs. **e** The water vapor flux of pure PEBAX membrane, PEBAX/F-Ce-4% membrane, and PEBAX/IL@F-Ce-4% membrane. **f** The effect of the pore diameter of nanosheets on water vapor permeability and selectivity of the MMMs. All error bars represent the standard deviation for at least five independent measurements.

Figure 5 shows the adsorption and diffusion of water vapor under different water vapor activities in pure PEBAX, PEBAX/F-Ce-4%, and PEBAX/IL@F-Ce-4% membrane. The water vapor adsorption in these membranes increases exponentially with the increase of water vapor activity, and the adsorption isotherm represents Flory-Huggins type adsorption^35^. The presence of hydrophilic functional groups in nanosheets tends to promote the affinity of MMMs for water molecules. It can be seen from Fig. 5a that the water vapor adsorption capacity of all MMMs is higher than that of pure membranes, which is more obvious in PEBAX/IL@F-Ce MMMs. The water vapor adsorption of the PEBAX/IL@F-Ce-4% membrane is significantly increased by 23.3% in comparison with the pure PEBAX membrane as the water vapor activity reaches saturation. This is attributed to the increased [DCA] anions ((-CN) in the [DCA] anions are characteristic groups that interact with the water molecules) from IL@F-Ce and the good interface compatibility between IL@F-Ce with the polymer matrix. The excellent solubility of water vapor on the surface of MMMs is beneficial to their permeability.

In Fig. 5b, the Fickian diffusion coefficient of PEBAX/F-Ce-4% MMM is higher than that of pure PEBAX matrix at different water vapor activity, indicating that the pore structure of F-Ce nanosheets plays a significant role in the diffusion of water vapor. In addition, the ILs in the IL@F-Ce nanosheets occupy part of the pores, resulting in rapid transmission of water vapor along the pores channel of the nanosheets. Hence, the Fickian diffusion coefficient of PEBAX/IL@F-Ce-4% MMM is improved further.

In addition, the water vapor adsorption kinetics test of the nanosheets is carried out at 30 °C and water vapor activity of 0.93 (Supplementary Fig. 4). Compared with F-Ce nanosheets, IL@F-Ce nanosheets show a higher adsorption rate and adsorption amount for water vapor due to the richer hydrophilic functional groups. Both F-Ce and IL@F-Ce nanosheets have high water vapor adsorption (Supplementary Table 2), which is related to their mesoporous structure, hydrophilic surfaces, and large specific surface area.

### Water vapor permeation performance of MMMs

To study the influence of the mesoporous nanosheets contents on the water vapor permeability and selectivity of the PEBAX matrix membrane, a mixed gas permeability test is carried out containing water vapor and N_2_ at 30 °C and 0.25 MPa, and water vapor activity of 0.93. As illustrated in Fig. 5c, the water vapor permeability and selectivity of MMMs all show a trend of first increase and then decrease with the increase of mesoporous nanosheets loading. At the F-Ce nanosheets loading of 4wt%, PEBAX/F-Ce MMM manifests the highest water vapor permeability (3.19 × 10^5^ Barrer) and H_2_O/N_2_ selectivity (1.46 × 10^5^), which are 67 and 57% higher than pure PEBAX membranes, respectively. The MMMs loaded with IL@F-Ce are improved more significantly than the MMMs loaded with F-Ce nanosheets. The water vapor permeability (4.53 × 10^5^ Barrer) and H_2_O/N_2_ selectivity (1.69 × 10^5^) reach the maximum values as the content of IL@F-Ce is 4%, which are 137 and 82% higher than pure PEBAX membranes, respectively. To explore the effect of [Emim][DCA] ILs in IL@F-Ce nanosheets on the performance of water vapor permeation and separation, PEBAX/[Emim][DCA] MMMs are fabricated, as shown in Supplementary Fig. 5, as the content of ILs increases, the structure of the membranes becomes more irregular and curled. As can be seen from Supplementary Fig. 6, due to the strong hygroscopicity of ILs in the PEBAX-based membrane, the water vapor permeability increases as the ILs loading increases, which indicates that ILs have a positive effect on the enhancement of water vapor permeability.

Besides, to probe the universality of IL@F-Ce nanosheets to improve the water vapor permeability of MMMs, Nafion/IL@F-Ce MMMs, and SPEEK/IL@F-Ce MMMs are fabricated, and the Ce element is regularly distributed on the cross-sectional image of MMMs (Supplementary Fig. 7). The water vapor permeability and selectivity are shown in Fig. 5d. It can be clearly seen that the introduction of a small amount of IL@F-Ce nanosheets effectively improves the performance of the Nafion and SPEEK membrane. When the loading amount of IL@F-Ce nanosheets is 4 wt%, the water vapor permeability and the H_2_O/N_2_ selectivity of the SPEEK/IL@F-Ce MMMs is 4.3 × 10^5^ Barrer and 0.8 × 10^6^, respectively, and when the loading amount of IL@F-Ce nanosheets is 5 wt%, the water vapor permeability and the H_2_O/N_2_ selectivity of the Nafion/IL@F-Ce MMMs is 5.3 × 10^5^ Barrer and 1.6 × 10^6^, respectively.

It is worth noting that the water vapor flux of the membranes loaded with 2D mesoporous nanosheets are significantly higher than that of the PEBAX membrane (Fig. 5e), the flux of the PEBAX membrane is 0.057 kg·m^−2^·h^−1^, and the water flux increases after introducing mesoporous nanosheets. The water fluxes of PEBAX/F-Ce-4% membrane and PEBAX/IL@F-Ce-4% membrane are 0.082 kg·m^−2^·h^−1^ and 0.11 kg·m^−2^·h^−1^, which are 44 and 93% higher than PEBAX membrane, respectively. It is obvious that PEBAX-based MMMs regulated mesoporous nanosheets have an exceptional selectivity on water vapor in the mixed gas.

According to our previous work, the pore diameter of the F-Ce nanosheets is adjusted by changing the pH value of the reaction system, and the result is shown in Supplementary Fig. 8 and Supplementary Table 3. Subsequently, F-Ce nanosheets with different pore diameters are introduced into the membrane at the load of 4 wt% to test their water vapor permeation and separation performance. The result is shown in Fig. 5f, the water vapor permeability of MMMs did not vary significantly with the increase in the average pore diameter of F-Ce nanosheets, while the water vapor permeability of MMMs suddenly increases to a maximum value of 3.84 × 10^5^ when the pore diameter of F-Ce nanosheets is 30.38 nm. Notably, the H_2_O/N_2_ selectivity decreases (from 1.78 × 10^5^ to 1.02 × 10^5^) as the average pore diameter of the nanosheets increases. Further, for comparison, GO nonporous nanosheets are introduced into the PEBAX matrix with the equal loading to prepare MMMs, and its properties are studied. As evident from Fig. 5f, the water vapor permeability of the PEBAX/GO membrane is lower than PEBAX/F-Ce membrane fabricated. It shows that the pore diameter of F-Ce nanosheets has a direct effect on the performance of water vapor permeability and selectivity. The effect of operation temperature on the permeation performance of the pure PEBAX, PEBAX/F-Ce-4%, and PEBAX/IL@F-Ce-4% membrane was investigated (Supplementary Fig. 9), and the permeability activation energies of the membranes were calculated (Supplementary Table 4).

**Fig. 6:**
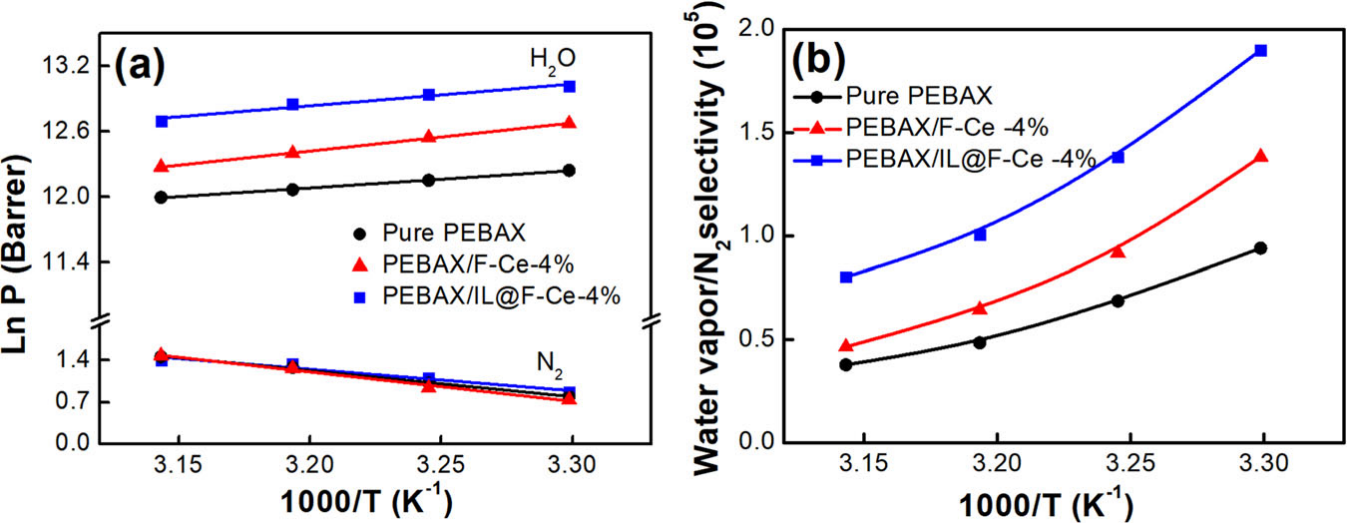
Effect of feed gas temperature. **a** Water vapor permeability coefficient (Ln P) and **b** selectivity toward H_2_O and N_2_ as a function of reaction temperature for the pure PEBAX membrane, PEBAX/F-Ce-4% membrane, and PEBAX/IL@F-Ce-4% membrane.

The effect of feed gas temperature on the water vapor permeation coefficient of the pure PEBAX, PEBAX/F-Ce-4%, and PEBAX/IL@F-Ce-4% membrane is displayed in Fig. 6a. It can be seen that *Ln P* has a linear relationship with *1/T*, and the influence of temperature on the gas permeability confirms to the Arrhenius Eq. (1):

1 $${In}\,P={In}\,{P}_{0}-\frac{{E}_{p}}{R}\left(\frac{1}{T}\right)$$

In the formula: *In P* is the permeation coefficient, *P*_*0*_ is the pre-exponential factors, *R* is the gas constant, *T* is the absolute temperature, and *E*_*p*_ is the activation energy of permeability.

Furthermore, as can be seen from Fig. 6b, the PEBAX/IL@F-Ce-4% membrane shows relatively higher H_2_O/N_2_ selectivity than pure membranes in the range of 30–45 °C. As the temperature rises from 30 °C to 45 °C, the selectivity of PEBAX/IL@F-Ce-4% membrane decreases from 1.89 × 10^5^ to 0.8 × 10^5^, and the selectivity of pure PEBAX membrane declines from 0.93 × 10^5^ to 0.37 × 10^5^. The increase in temperature can accelerate the movement of gas molecules and promote the diffusion of gas molecules in the membrane. On the other hand, the solubility of gases decreases with increasing temperature. As shown in Supplementary Fig. 9, the water vapor permeability of all membranes decreases as the temperature increases, indicating that the transport of water vapor in the MMM is mainly controlled by the solubility. The solubility of gas molecules on the solid surface is generally an exothermic process (the enthalpy of adsorption is negative), and thus the solubility decreases as the temperature rises. For N_2_, the permeability increases with increasing temperature, indicating that its transport in the membrane is mainly controlled by the diffusion process^9^. From Supplementary Table 4, the water vapor activation energy *E*_*P*_ of permeability is negative, and the N_2_ activation energy *E*_*P*_ of permeability is positive, verifying the above-mentioned theory. The absolute value of gas activation energy of permeability of all MMMs is higher than that of pure PEBAX membrane because the introduction of F-Ce nanosheets reduces the molecular chain packing efficiency of MMMs, increases flexibility, and gas permeability is greatly affected by temperature.

**Fig. 7:**
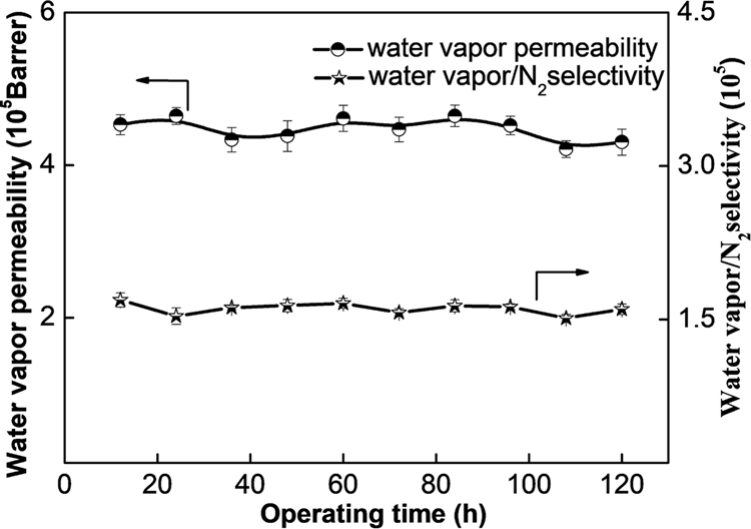
Stability analysis. The water vapor permeation stability of PEBAX/IL@F-Ce-4% membrane. The experimental conditions at 30 °C and 0.25 MPa, and water vapor activity of 0.93. All error bars represent the standard deviation for at least five independent measurements.

The stability of the membrane is very important for practical applications. As illustrated in Fig. 7, the water vapor permeability of the PEBAX/IL@F-Ce-4% membrane varies within the error range during the 120 h test, while the selectivity remains almost unchanged. The PEBAX/IL@F-Ce-4% MMM exhibits superior long-term stability and separation performance.

### Construction of water vapor transport channels

**Fig. 8:**
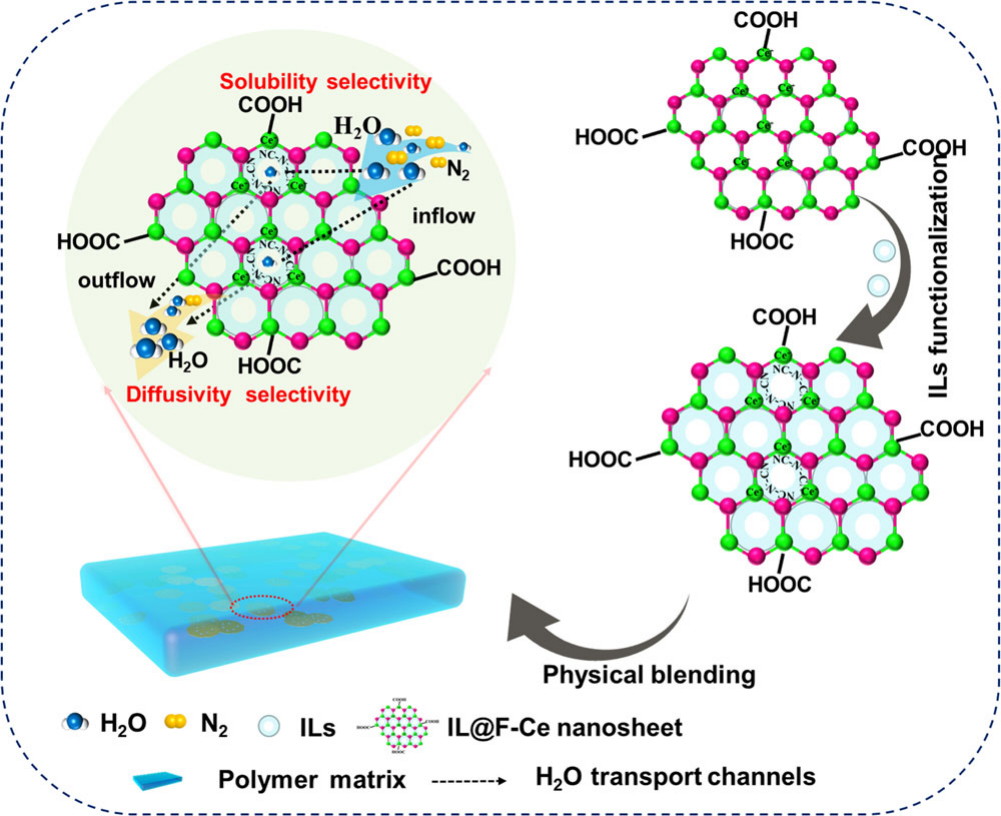
Construction of the transport channels. Schematic diagram of the water vapor transport channels in MMMs regulated by 2D mesoporous nanosheets.

2D mesoporous nanosheets play an important role for the construction of water vapor transport channels in MMMs. The schematic diagram of the transport channels of water vapor in MMMs regulated by 2D mesoporous nanosheets is shown in Fig. 8. F-Ce nanosheets have a good affinity for water molecules due to the large surface area and -COOH functional group, which can fast drive water molecules to transport through the membrane. Moreover, the slit-shaped mesoporous structure of F-Ce nanosheets provides transport channels for water molecules, which promotes water molecules permeation but blocks N_2_, because the competitive occupancies of water vapor would narrow the effective transport channels with impaired diffusion for N_2_. When the composite of ILs and F-Ce nanosheets, the ILs on the surface of nanosheets adsorb preferentially water vapor due to their strong hygroscopicity, thereby improving the water vapor solubility selectivity. On the other hand, the ILs present in the pores of the nanosheets provide well-defined transport channels for water vapor, thereby improving the water vapor diffusion selectivity. The diffusion mechanism and solubility mechanism of the membranes are strengthened simultaneously.

**Fig. 9:**
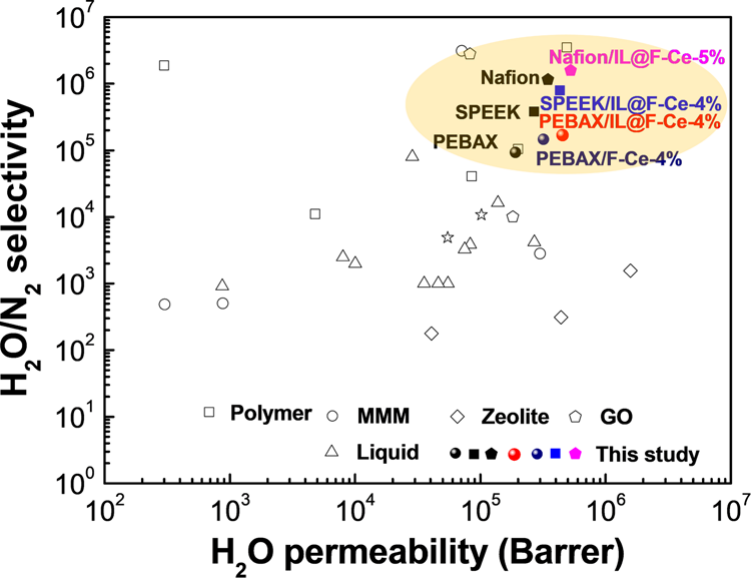
Performance comparison. Comparison of current results with those of literature for similar systems in a permeability and H_2_O/N_2_ selectivity plot.

To provide further support for function of these transport channels, the water vapor permeability of MMMs that are being investigated are listed in Supplementary Table 5 and compared with the properties of the MMMs regulated by 2D mesoporous nanosheets. It can be seen from Fig. 9 that the performance of the fabricated membrane in this study reached one of the highest levels currently reported in the literature. Furthermore, PEBAX/GO MMM is taken as an example (Supplementary Fig. 10), the water vapor permeability of PEBAX/GO MMM is only increased by 32% compared with pure membrane. Although the GO nanosheets increase the water molecule adsorption of the base membrane due to the presence of abundant hydrophilic groups, the high aspect ratio restricts more the diffusion of gas molecules. However, F-Ce and IL@F-Ce nanosheets have the advantages of mesoporous structure and high specific surface area, which provides not only abundant adsorption sites for water molecules but also fast diffusion channels, so that the water vapor permeability of IL@F-Ce/PEBAX MMMs is almost 2 fold higher than that of the GO/PEBAX MMMs at equal loading (4wt%). Therefore, 2D mesoporous nanosheets provide an attractive prospect in the field of industrial dehumidification and humidification.

## Conclusions

In this study, a simple and economical method is used to prepare 2D F-Ce nanosheets and ionic liquid-functionalized F-Ce nanosheets. The MMMs based on these 2D nanosheets significantly improve the permeability and selectivity of water vapor. The hydrophilic functional groups of the F-Ce nanosheets with high specific surface area have a strong affinity for water vapor, thereby enhancing the water vapor adsorption capacity. Moreover, the slit-shaped mesoporous structure of the F-Ce nanosheets provides transport channels for water molecules and elevate the water vapor diffusivity. IL@F-Ce nanosheets have higher water vapor adsorption and rapid diffusion channels than the former, and thus it is more prominent in terms of water vapor permeability. The MMMs containing 4 wt.% IL@F-Ce nanosheets have the highest water vapor permeability (4.53 × 10^5^ Barrer) and H_2_O/N_2_ selectivity (1.69 × 10^5^). This research provides opportunities for the dehumidification and humidification process of membrane technology.

## Methods

### Materials

PEBAX® 1074 (ARKEMA) was obtained from Tianjin Kelaite Trade Co., Ltd. (China). Graphene oxide was purchased from Nanjing XFNANO Materials Technology Co., Ltd. (China). Alcohol-based Nafion dispersion (5wt%) was purchased from DuPont. Poly(ether ether ketone) was purchased from Victrex (United Kingdom), Ltd. n-butanol (99.5%) and methanol (99.5%) was obtained from Tianjin Kemiou Chemical Reagent Co., Ltd. Cerium acetate (99.9%) and sodium fluoride (99.9%) were obtained from Shanghai Aladdin Biochemical Technology Co., Ltd. (China). 1-Ethyl-3-methylimidazolium dicyanamide ([EMIM][DCA]) ionic liquid was obtained from Shanghai Moni Chemical Co., Ltd. The chemical structures of PEBAX^®^ 1074 and [EMIM][DCA] were displayed in Supplementary Fig. 11. Nitrogen (99.99%) and helium (99.99%) were obtained from Tianjin Huanyu gas Co., Ltd. (China).

### Synthesis of F-Ce nanosheets^32^

In detail, cerium acetate (36 mmol) formed nanoparticles through a hydrolysis reaction in an aqueous solution at pH = 7. In the process of dripping sodium fluoride solution (100 ml 1.2 mg·ml^−1^), the fluorine atoms were exchanged with the oxygen atoms in the hydrolyzed nanoparticles, resulting in changes in the structure of the nanoparticles and carried more positive charges, while acetate acted as a surfactant and binder on the surface of the particles. Under the action of electrostatic interaction and hydrogen bonding, the nanoparticles were assembled into F-Ce nanosheets through the attachment process^36^.

### Synthesis of IL@F-Ce nanosheets

To improve the hydrophilicity of F-Ce nanosheets, IL@F-Ce was prepared by soaking F-Ce in a [EMIM][DCA] water solution (ILs and F-Ce nanosheets (1:10 in weight)). Subsequently, the IL@F-Ce product was obtained by centrifuging (8000 rpm) treatment. The IL@F-Ce product functionalized by the ionic liquid was washed several times with pure water, obtaining IL@F-Ce nanosheets.

### Preparation of PEBAX-based MMMs

To fabricate the membrane casting solution, PEBAX^®^ 1074 was dissolved in the n-butanol solvent at 90 °C under stirring over 4 h until a uniform solution was obtained. The nanosheets were initially dispersed in a certain amount of n-butanol solvent, stirring at room temperature for 2 h, and then were put into a 250 W ultrasonic cleaner, ultrasonically treated for 2 h to achieve sufficient dispersion. The nanosheets suspension were mixed with the PEBAX solution, and the resulting solution mixture was continuously stirred for 4 h and then subjected to ultrasonic treatment. Finally, the mixed solution was cast onto a glass plate and treated in an oven at 50 °C for overnight, and then further dried under vacuum to remove trace of n-butanol solvent. The concentration of PEBAX polymer was 5wt.%, and the mass percentage of F-Ce or IL@F-Ce nanosheets in the final membranes was changed from 0.5 wt.% to 10 wt.%. For better comparison, Nafion-based MMMs, SPEEK-based MMMs and PEBAX/GO MMMs were also prepared. (The detailed preparation process can be found in the Supplementary Methods).

### Characterization

Field emission scanning electron microscope (FESEM, Hitachi S4800) was used to observe the morphology of F-Ce and IL@F-Ce nanosheets, and MMMs. The morphology of F-Ce and IL@F-Ce nanosheets was studied through a transmission electron microscope (TEM, Hitachi H7650), and the element distribution of the functionalized F-Ce nanosheets was studied by EDS mapping. The chemical structure of the F-Ce and IL@F-Ce nanosheets were characterized by Fourier transform infrared (FTIR, Bruker, vertex80). The surface chemical composition of the unwashed, washed, and ultrasonic cleaned IL@F-Ce nanosheets were analyzed by X-ray photoelectron spectrometer (XPS, Thermofisher, K-alpha). The specific surface area and pore size were analyzed by an automatic physical adsorption instrument (BET, Quantachrome, Autosorb-iQ-C). The crystal structures of F-Ce and IL@F-Ce nanosheets were studied by X-ray diffractometer (XRD, Bruker, D8 discover). STA409PC thermogravimetric analyzer in N_2_ atmosphere was used to analyze the thermal properties of the nanosheets. The zeta potential of F-Ce and IL@F-Ce nanosheets in water and n-butanol was tested using a nanoparticle size zeta potentiometer (Litesizer 500) to measure. To characterize the glass transition temperature (Tg) of MMMs, which was explored by DSC200F3 differential scanning calorimeter. The water contact angle (WCA) was measured by the JC2000C meter to study the hydrophilicity of MMMs.

The solubility of water vapor in a membrane can be evaluated by the change in the weight of the membrane caused by the variation in its adsorption in the vapor phase. Therefore, The equilibrium adsorption capacity (*C*) of water vapor can be calculated according to the Eq. (2):

2 $$C=\frac{{M}_{{{{{{\rm{\infty }}}}}}}{-M}_{{dry}}}{{M}_{{dry}}}$$

Where *M*_*∞*_ [g] was referred to as the equilibrium mass of the membrane sample and the absorbed water at a given water vapor activity, and *M*_*dry*_ [g] was the dry weight of the membrane.

The diffusion coefficient of water vapor can be calculated from the kinetic adsorption data. Based on the Fickian diffusion model and the equation was as follows (Eq. (3)):

3 $${ln}\left(1-\frac{{M}_{t}}{{M}_{{{{{{\rm{\infty }}}}}}}}\right)={ln}\frac{8}{{\pi }^{2}}-\frac{{\pi }^{2}{Dt}}{{l}^{2}}$$

Where *M*_*t*_ (g) and *M*_*∞*_ (g) were the mass of adsorbed gas-phase molecules at time t and at equilibrium, respectively, *l* [m] was the membrane thickness (For comparison, we assume that the membrane thickness is constant, although it will increase due to the swelling), and *D* (m^2^·s^−1^) was the diffusion coefficient. In the short time region (*M*_*t*_/*M*_*∞*_ < 0.5), the equation was as follows^37,38^ (Eq. (4)):

4 $$\frac{{M}_{t}}{{M}_{{{{{{\rm{\infty }}}}}}}}=\frac{4}{l}\sqrt{\frac{{Dt}}{\pi }}$$

### Gas permeability measurements

The schematic diagram of the gas dehumidification experimental device is shown in Supplementary Fig. 12. The operating temperature was controlled by the oven. The nitrogen stream was passed through the humidification tank, and the humidification of the nitrogen/steam binary mixture was adjusted by the mass flow controller (Sevevstar, Model D07-7). Subsequently, the binary nitrogen/steam was passed through a gas-liquid separation device to remove the small droplets contained therein, and finally, the mixed gas was continuously fed into the membrane cell at a flow rate of 600 ml min^−1^. The operating temperature of the membrane cell could be regulated within the range of 25 to 45 °C. After helium as a purge gas passed through the membrane cell, the components on the permeate side were analyzed. The moisture content in the upstream and downstream of the membrane module was measured by a dew-point meter (Vaisala, Model DM70). The water vapor on the permeate side was immersed in a cold trap to condense, and its permeability was calculated by weighing the permeated vapor gathered in the condenser. The nitrogen content of the feed and permeate streams was analyzed by gas chromatography (GS, Model 101 N).

The permeate flux, permeability, and selectivity were calculated using the following equation^39^, respectively:

5 $${J}_{m}=\frac{{W}_{p}}{{At}}$$

6 $${P}_{i}=\frac{{\varphi }_{v,{tot}}\times F}{A}\times \frac{l}{{P}_{r}-{P}_{p}}$$

7 $$\alpha =\frac{{P}_{i}}{{P}_{j}}$$

Where *J*_*m*_ was the permeate flux [kg·m^−2^·h^−1^], *P*_*i*_ was the permeability of species i [barrer] (1 barrer = 1 × 10^−10^ cm^3^(STP)·cm·cm^−2^·s^−1^·cmHg^−1^), *α* was the selectivity of species i to j, *W*_*p*_ was the amount of liquid condensed in the cold trap, *A* was the surface area of the membrane, *t* was the testing duration time to collect *W*_*p*_, *φ*_*v,*tot_ was the volume flow of He at permeate side, *F* represented the volume fraction of the N_2_ present in He flow, *l* was the membrane thickness, *p*_*r*_ and *p*_*p*_ were referred to as the feed and sweep side partial pressures of the measured gas, respectively.

## Supplementary information

Revised Supporting information

## Data Availability

All the relevant data of this study are available within this paper and its Supplementary Information are available from the corresponding author upon reasonable request.
