# Supplementary material for: Constructing rapid water vapor transport channels within mixed matrix membranes based on two-dimensional mesoporous nanosheets
Source: Commun Chem. 2022 May 24;5:65. doi: 10.1038/s42004-022-00681-9 (PMC9814085; doi:10.1038/s42004-022-00681-9)
Supplement: Supplementary file 1 — Revised Supporting information [file 42004_2022_681_MOESM1_ESM.pdf]

Supplementary Information

Constructing rapid water vapor transport channels within mixed matrix membranes based on two-dimensional mesoporous nanosheets

Fengkai Wang[1], Shurui Han[1], Yanli Zhang[1], Lei Gao[1], Xu Li[1], Lizhi Zhao[1], Hui Ye[1], Hong Li[1]
Qingping Xin[1*] & Yuzhong Zhang[1*]

[1]State Key Laboratory of Separation Membranes and Membrane Processes, School of Materials Science and Engineering, Tiangong University, Tianjin 300387, China

Corresponding authors* E-mail:

xinqingping@tiangong.edu.cn (Qingping Xin)

zhangyz2004cn@vip.163.com (Yuzhong Zhang)

Table of Contents

1. Supplementary Methods ••3-4

2. Supplementary Figs 1-12••5-10

3. Supplementary Tables 1-5••11-13

4. Supplementary References•••13-14

Supplementary Methods

Preparation of Nafion-based MMMs

Nafion-based MMMs were prepared by solution casting method. A certain amount of F-Ce or IL@F-Ce nanosheets were added into the Nafion dispersion. Vigorous stirring and ultrasonic treatment were successively applied to obtain a uniform mixture. Then, the mixed solution was cast on a glass plate at 50°C overnight. The content of F-Ce or IL@F-Ce nanosheets was changed from 0.5 wt.% to 10 wt.%.

Preparation of SPEEK

10 g of PEEK was dried in a 100 ◦C vacuum oven for 72 h and dissolved in 100 ml of H_2SO_4 (98 wt%). The solution was vigorously stirred at room temperature during 24 h, and then heated up to 50 ℃ for 4 h. Then, the solution was slowly poured into lots of ice-cold water under continuous agitation. The precipitate was filtered and washed several times with deionized water until pH＞5, and then dried in a vacuum oven at 50 ℃ for 48 h. The sulfonated degree of SPEEK was 60 %.

Preparation of SPEEK-based MMMs

First, SPEEK was dissolved in methanol at room temperature. An amount of of F-Ce or IL@F-Ce nanosheets was dispersed in methanol and the resulting suspension was added into SPEEK- methanol solution under vigorously stirring and ultrasonic treatment to get a fine dispersion of nanosheets in the polymer solution. After degasification, the mixture was poured on a clean glass plate and heated at 50 °C overnight. And, the membranes were peeled off from the glass plate, dried under vacuum at 60 °C for 24 h to remove trace of methanol solvent. The concentration of SPEEK polymer was 15 wt.%, and the content of F-Ce or IL@F-Ce

nanosheets was changed from 0.5 wt.% to 10 wt.%.

Preparation of PEBAX/GO MMMs

The PEBAX/GO MMMs was prepared by the same manner as that for PEBAX-based MMMs, and the mass percentage of GO nanosheets in the final membranes was 4 wt.%.

Supplementary Figs

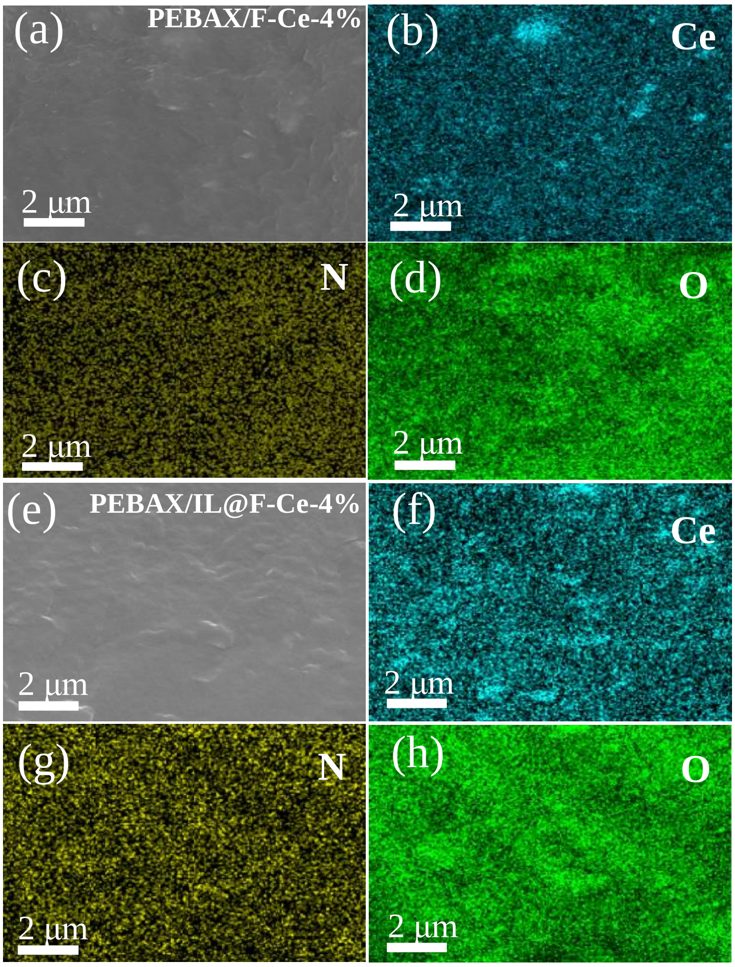

Supplementary Fig. 1 SEM images (a, e) and element mappings of Ce (b, f), N (c, g), and O (d, h) element distributions for the PEBAX-based MMMs.

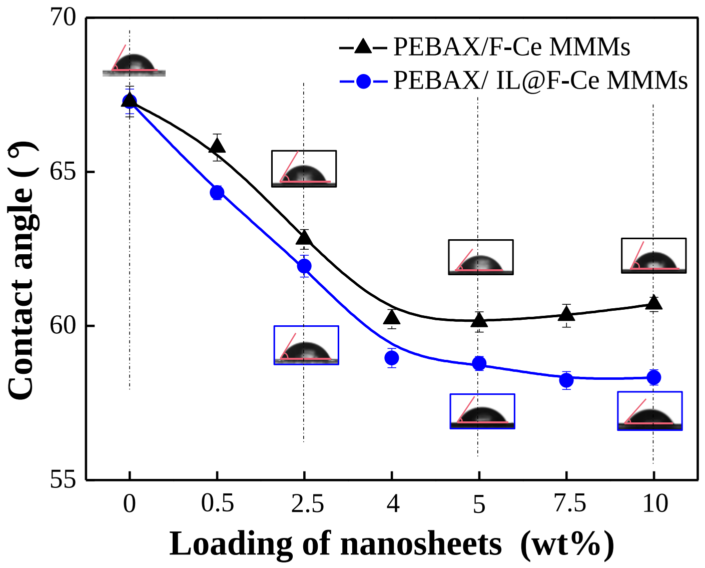

Supplementary Fig. 2 Water contact angle of F-Ce and IL@F-Ce nanosheets incorporated MMMs

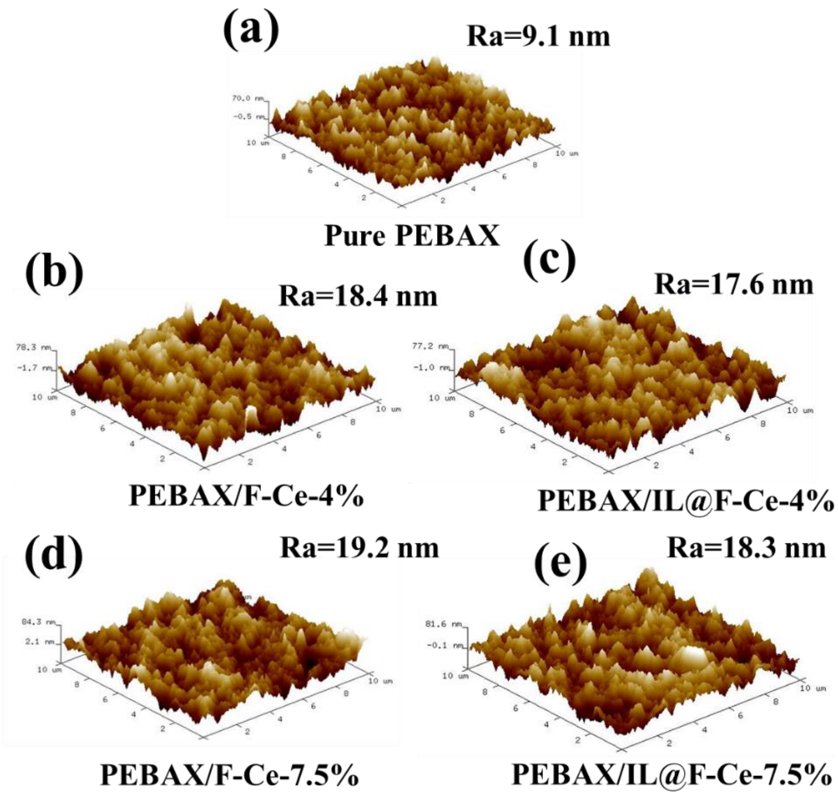

Supplementary Fig. 3 The three-dimensional AFM images and roughness parameters of (a) the pure PEBAX membrane and MMMs incorporated with 4 wt.% (b), and 7.5 wt.% (c) F-Ce nanosheets and 4 wt.% (d), and 7.5 wt.% (e) IL@F-Ce nanosheets

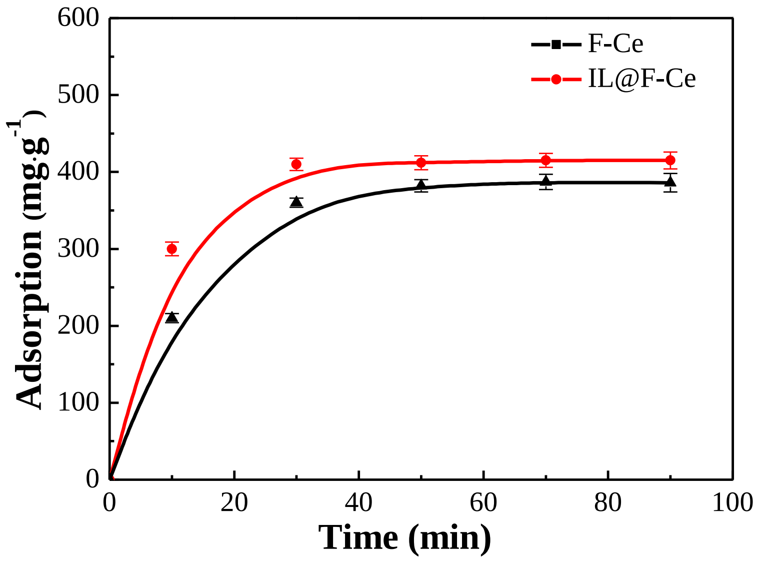

Supplementary Fig. 4 Kinetics of water vapor adsorption for F-Ce, and IL@F-Ce nanosheet

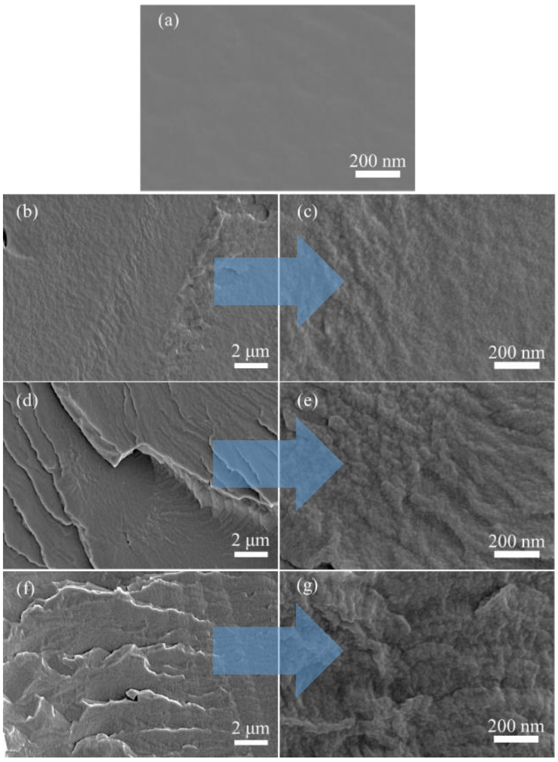

Supplementary Fig. 5 Cross-sectional SEM images of (a) Pure PEBAX, (b, c) PEBAX/IL (2.5 wt.%), (d, e) PEBAX/IL (4 wt.%), (f, g) PEBAX/IL (10 wt.%)

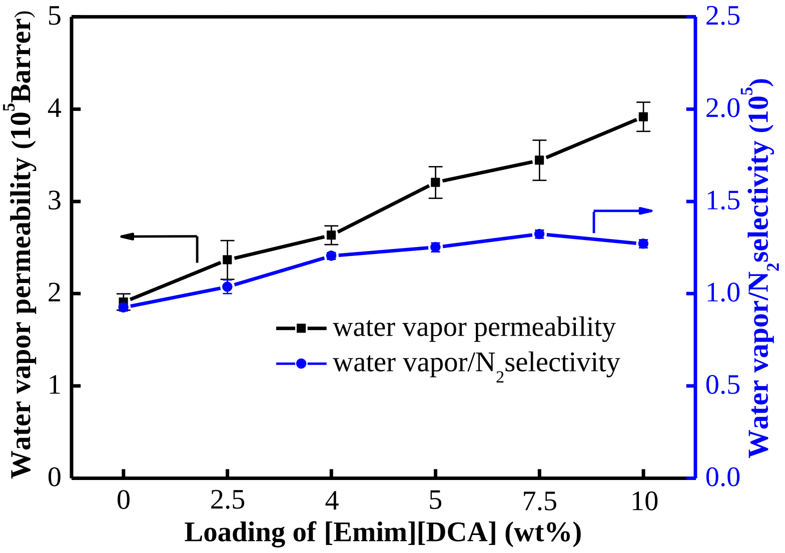

Supplementary Fig. 6 The water vapor permeability and water vapor/N_2 selectivity of the PEBAX/[Emim][DCA] MMMs.

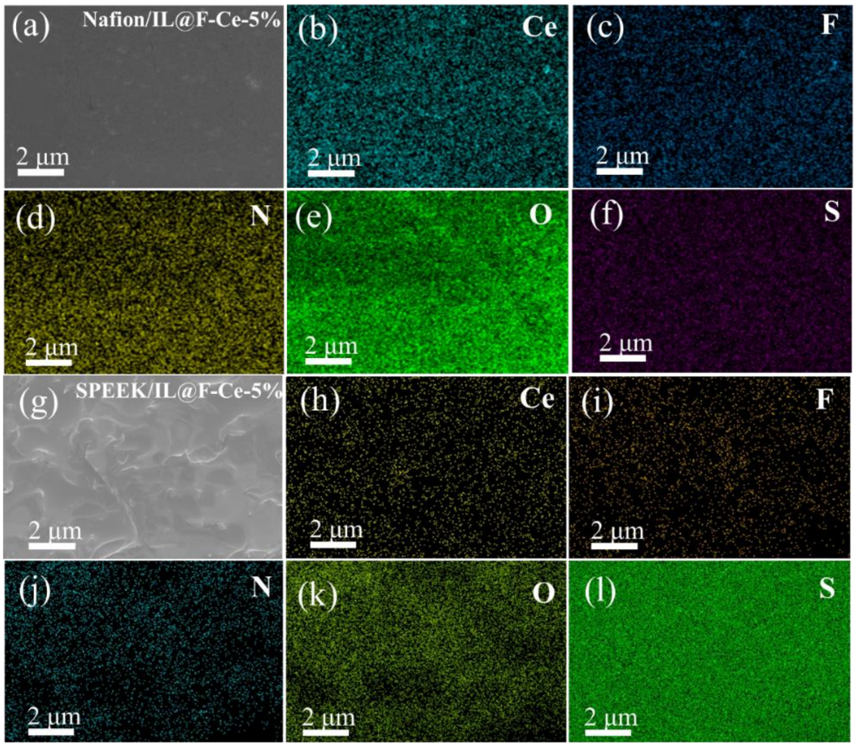

Supplementary Fig. 7 Surface SEM images (a, g) and element mappings of Ce (b, h), F (C, i), N (d, j), O (e, k), and S (f, l) element distributions for the Nafion-based M MMs and SPEEK-based MMMs, respectively.

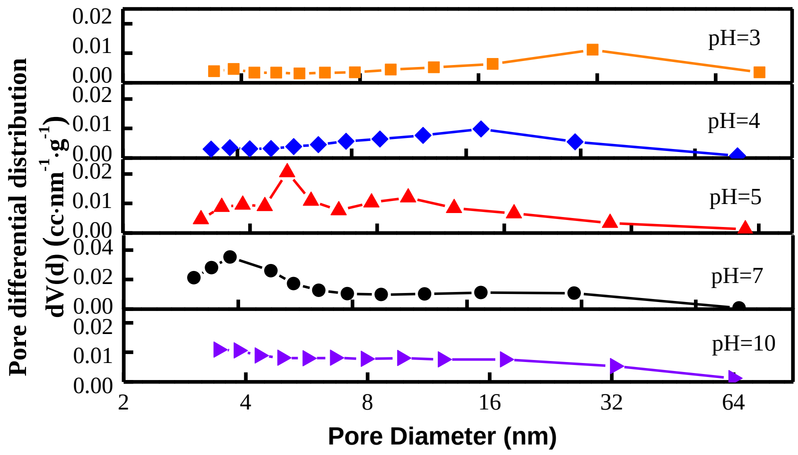

Supplementary Fig. 8 Pore diameter distribution of F-Ce nanosheets synthesized at different initial pH

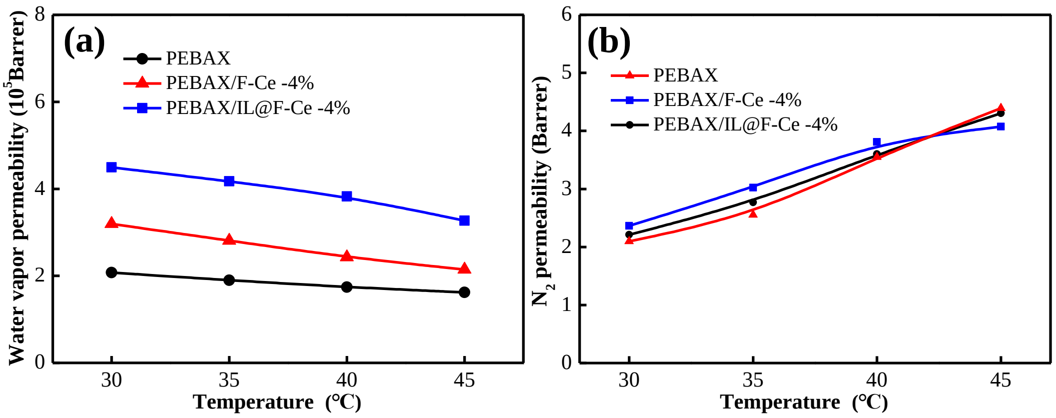

Supplementary Fig. 9 Temperature variation of the permeability toward (a) water vapor and (b) N_2 for the pure PEBAX, and the MMMs.

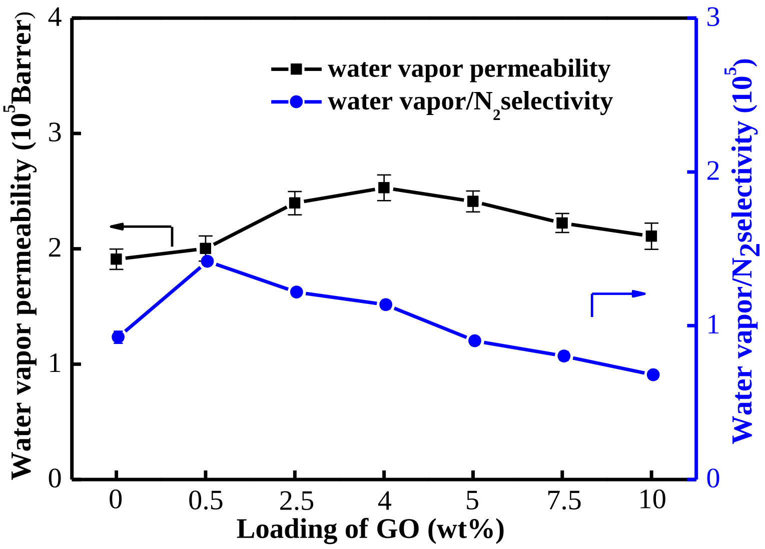

Supplementary Fig. 10 The water vapor permeability and water vapor/N_2 selectivity of the PEBAX/GO MMMs.

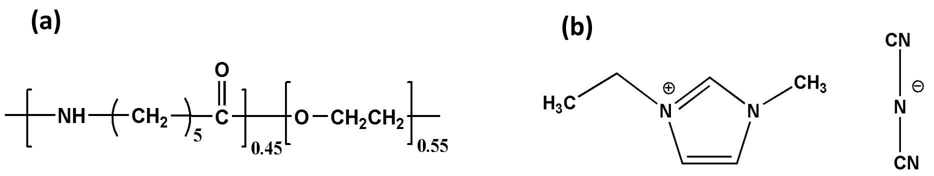

Supplementary Fig. 11 Chemical structures of (a) PEBAX® 1074 and (b) [EMIM][DCA]

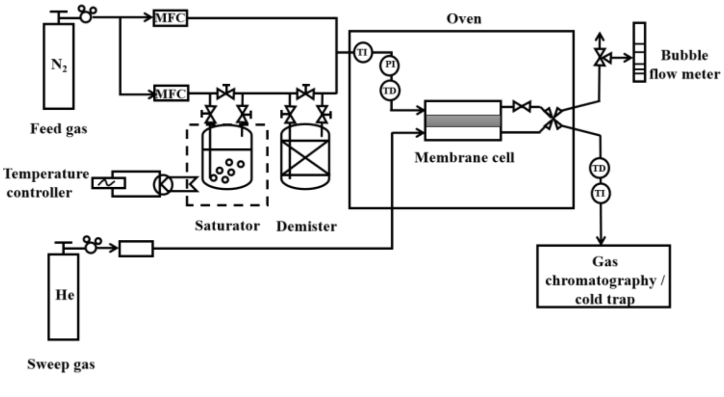

Supplementary Fig. 12 Schematic diagram of the device for measuring the permeability of water vapor/N_2 mixture

Supplementary Tables

Supplementary Table 1. XPS surface element analysis of IL@F-Ce nanosheets

Treatment	F (Atomic %)	Ce (Atomic %)	O (Atomic %)	N (Atomic %)
/	11.36	4.82	17.33	12.39
washing	22.4	10.21	21.71	3.28
washing, ultrasonic cleaning	23.97	10.35	20.94	3.31

Supplementary Table 2. The saturated adsorption amount and adsorption equilibrium time of water vapor adsorption for F-Ce, and IL@F-Ce nanosheets

Samples	saturated adsorption amount	adsorption equilibrium time
F-Ce nanosheets	387 $mg \cdot g^{-1}$	~ 40 min
IL@F-Ce nanosheets	415 $mg \cdot g^{-1}$	~ 30 min

Supplementary Table 3. Pore structure parameters of F-Ce nanosheets synthesized at different initial pH

pH	Average pore size (nm)	Surface area (m^2 g^{-1})	Pore volume (cm^3 g^{-1})
3	30.38	68.75	0.60
4	17.50	52.24	0.30
5	4.89	83.74	0.26
7	3.81	162.75	0.52
10	3.42	76.46	0.31

Supplementary Table 4. Activation energy of the permeability for the pure PEBAX membrane, PEBAX/F-Ce-4% membrane, and PEBAX/IL@F-Ce-4% membrane

Membrane	E_P Value (KJ mol^{-1})	
	N_2	H_2O
PEBAX	36.3±0.3	-13.3±1.1
PEBAX/F-Ce-4%	40.9±0.5	-19.6±1.2
PEBAX/IL@F-Ce-4%	38.6±0.3	-28.6±1.5

Supplementary Table 5. The comparison of membrane separation performance

**Sample**	**Temperature (℃)**	**Thickness (μm)**	**H_2O permeability (Barrer)**	**H_2O/N_2 selectivity**	**Ref.**
PEO-PBT	30	30	85500	40500	[1]
PAN	30	12.7	300	1875000	[2]
PEBAX1074	30	78	200000	104167	[3]
SPEEK	30	51	490000	350000	
TMSC	23	1.2	4808	11000	[4]
PVA/LiCl	24	180	300000	2800	[5]
PEBAX1657/GO	21	5.7	28632	80000	[6]
PSf/Si-TFN	30	0.4	881	501	[7]
PSf/$D^{1.0}T^{0.2}cTiO_2^{0.2}$	30	0.226	303	486	[8]
ABn-NH-TFN	30	0.31	872	913	[9]
PBI/TiO_2	22	20	71000	3100000	[10]
Zeolite NaA	32	2	40664	178	[11]
GO	30.8	6	182000	10000	[12]
GLX-cross-linked GO	/	1.64	82380	2800000	[13]
[C_2MIM][Tf_2N]	31	132	82883	3843	[14]
[C_2MIM][BF_4]	31	132	138138	16300	
[N_{4111}][Tf_2N]	31	132	74989	3290	
TEG	30	47	8010	2500	[15]

Supplementary Table 5. (continued)

PEG-400	22	67	10017	2000	
[C_2MIM][DCA]	25	11.5	55016	> 1000	
[C_2MIM][ESU]	25	10.3	46196	> 1000	[16]
[C_2MIM][BF_4]	25	9.9	35521	> 1000	
TEG	25	12.8	27556	> 1000	
UiO-66-NH_2	22	~2	440000 ± 120000	312 ± 68	[17]
IL@UiO-66-NH_2	22	~2	1580000 ± 110000	1564 ± 218	
[C_4MIM][Br]	22	~58	270000 ± 90000	4206 ± 1182	
PEBAX	30	105	190000	92500	This work
PEBAX/F-Ce-4%	30	111	318000	146000	This study
PEBAX/IL@F-Ce-4%	30	111	453000	169000	This work
Nafion	30	80	346000	1150000	This work
Nafion/IL@F-Ce-5%	30	85	525000	1580000	This work
SPEEK	30	90	268000	381000	This work
SPEEK/IL@F-Ce-4%	30	97	433000	796000	This work

Supplementary References

1 Sijbesma, H. et al. Flue gas dehydration using polymer membranes. J. Membr. Sci. **313**, 263-276 (2008).

2 Metz, S. et al. Transport of water vapor and inert gas mixtures through highly selective and highly permeable polymer membranes. J. Membr. Sci. **251**, 29-41 (2005).

3 Allen, S. M. et al. The barrier properties of polyacrylonitrile. J. Membr. Sci. **2**, 153-163 (1977).

4 Puspasari, T. et al. High dehumidification performance of amorphous cellulose composite membranes prepared from trimethylsilyl cellulose. J. Mater. Chem. A. **6**, 9271-9279 (2018).

5 Bui, D. T. et al. Water vapor permeation and dehumidification performance of poly(vinyl alcohol)/lithium chloride composite membranes. J. Membr. Sci. **498**, 254-262 (2016).

6 Akhtar, F. H. et al. Pebax®1657/Graphene oxide composite membranes for improved water vapor separation. J. Membr. Sci. **525**, 187-194 (2017).

7 Baig, M. I. et al. Synthesis and characterization of thin film nanocomposite membranes incorporated with surface functionalized Silicon nanoparticles for improved water vapor permeation performance. Chem. Eng. J. **308**, 27-39 (2017).

8 Baig, M. I. et al. Development of carboxylated TiO_2 incorporated thin film nanocomposite hollow fiber membranes for flue gas dehydration. J. Membr. Sci. **514**, 622-635 (2016).

9 Akhtar, F. H. et al. Polybenzimidazole-based mixed membranes with exceptional high water vapor permeability and selectivity. J. Mater. Chem. A. **5**, 21807-21819 (2017).

10 Ingole, P. G. et al. Thin film nanocomposite (TFN) hollow fiber membranes incorporated with functionalized acid-activated bentonite (ABn-NH) clay: towards enhancement of water vapor permeance and selectivity. J. Mater. Chem. A. **5**, 20947-20958 (2017).

11 Xing, R. et al. Advanced thin zeolite/metal flat sheet membrane for energy efficient air dehumidification and conditioning. Chem. Eng. Sci. **104**, 596-609 (2013).

12 Shin, Y. et al. Graphene oxide membranes with high permeability and selectivity for dehumidification of air. Carbon **106**, 164-170 (2016).

13 Hung, W. S. et al. Tuneable interlayer spacing self-assembling on graphene oxide-framework membrane for enhance air dehumidification. Sep. Purif. Technol. **239**, 116499 (2020).

14 Scovazzo, P. Testing and evaluation of room temperature ionic liquid (RTIL) membranes for gas dehumidification. J. Membr. Sci. **355**, 7-17 (2010).

15 Ito, A. Dehumidification of air by a hygroscopic liquid membrane supported on surface of a hydrophobic microporous membrane. J. Membr. Sci. **175**, 35-42 (2000).

16 Kudasheva, A. et al. Dehumidification of air using liquid membranes with ionic liquids. J. Membr. Sci. **499**, 379-385 (2016).

17 Park, S. et al. Highly H_2O permeable ionic liquid encapsulated metal-organic framework membranes for energy-efficient air-dehumidification. J. Mater. Chem. A. **8**, 23645-23653 (2020).
